# Editorial: Complexity and Self-Organization

**DOI:** 10.3389/frobt.2021.668305

**Published:** 2021-03-26

**Authors:** Carlos Gershenson, Daniel Polani, Georg Martius

**Affiliations:** ^1^Departamento de Ciencias de la Computación, Instituto de Investigaciones en Matemáticas Aplicadas y en Sistemas, Universidad Nacional Autónoma de Mexico, Mexico, Mexico; ^2^Centro de Ciencias de la Complejidad, Universidad Nacional Autónoma de Mexico, Mexico, Mexico; ^3^Lakeside Labs GmbH, Klagenfurt, Austria; ^4^School of Engineering and Computer Science, University of Hertfordshire, Hatfield, United Kingdom; ^5^Autonomous Learning Group, Max Planck Institute for Intelligent Systems, Tübingen, Germany

**Keywords:** complexity, self-organization, formal methods, cognitive science, global issues

Complexity occurs when relevant interactions prevent the study of elements of a system in isolation. These interactions between elements may lead to the self-organization of the system. A system can be described as self-organizing when its global properties are a product of the interactions of its components. Complexity and self-organization are prevalent in a broad variety of systems. Because of this, they have been studied from multiple perspectives and disciplines, leading naturally to transdisciplinary studies.

The scientific study of complexity and self-organization was limited before the popularization of computers in the 1980s, as previous tools were insufficient to deal with hundreds or thousands of variables. Thus, computer science has been essential for these studies.

In computational intelligence, complexity and self-organization have been studied and exploited with different purposes. The aim of this Research Topic was to bring together novel research into a coherent collection, spanning from theory and methods to simulations and applications.

For example, it has been observed that complex systems studied by different disciplines reach a balance between change and stability that has been also described as criticality. This balance allows the maintenance of functionality (robustness) and also the potential to change in response to new situations (adaptability). The different contributions included in this Research Topic illustrate how this balance is present in a broad variety of phenomena.

We received 22 submissions, out of which ten were accepted.

Of these, three dealt with **formal methods** as a crucial tool for the understanding of complex and self-organizing systems. Voit and Meyer-Ortmanns propose a data-based approach to automatically infer heteroclinic networks. These are networks where nodes represent saddle fixed points in phase space, while edges are heteroclinic orbits (which occur when the unstable manifold of a saddle fixed point intersects the stable manifold of another saddle). The proposed method is based on a template system that uses a learning algorithm to adjust the eigenvalues at the saddles, eventually reconstructing the topology of the original heteroclinic network. This approach is promising to infer a structure that reproduces an observed function or dynamics.

Camargo uses an agent-based model of ideological alignment to explore the usefulness of different approaches and methods to analyze its dynamics. Camargo generalizes to argue that the proposed approaches can be applied to other agent-based models of social behavior, where complexity is such that measurements of the performance of the model are not explicit nor straightforward.

An obstacle of measurements is the lack of proper metrics. In this respect, Correa presents a mini-review on how metrics of emergence, self-organization, and complexity can contribute to commerce/consumer studies, in particular, to the understanding “electronic word-of-mouth” (EWOM) data. These metrics can be used as proxies to the degree of customers' comments diversification, customers' comments polarization, and the diversification-polarization balance.

A particular subset of the studies of complex systems concerns itself with issues of **cognitive science**, which is addressed in three of the papers. The first of them asks what happens when humans interact with humanoid robots that mimic their behavior in a self-organizing way. This interesting question is investigated in Mazumori et al. In their system, the robot's behavior is partially self-organized using a memory of prior interaction with other humans (art gallery visitors) and an internal dynamics that makes the robot only partially predictable. Engagement of the interaction partner and the inversion of roles, i.e., that the human starts to imitate the robot, are frequently observed.

Ramos-Fernandez et al. consider the question how information is being acquired and distributed in a group of individuals, specifically validated against data obtained from spider monkey colonies. The particular dynamics studied are the dynamics of subgroups of monkeys which split and merge (fission-fusion dynamics) and how these decisions are taken by integrating events experienced by the individuals over time. The different timescales of the dynamics (fights, signaling, and rank) are used by the faster degrees of freedom as reference in a form of “downward causation.” Thus, the decisions of the individuals of the collective make up the structure which, in turn, influences the decisions of the individuals.

Considering the neural mechanisms of behavior, Morales and Froese contribute to this research topic with a short study on the role of unsupervised learning in the formation of functional clusters in the *C. elegans* nervous system. Unsupervised Hebbian learning can be a self-optimization process to bring the initial network into a state of better generalization to new patterns.

Another important application of the study of complexity is the overarching field of **global issues** which is addressed in the following two papers. Nowhere does the importance of complexity science show as clearly as in issues of global ecological and economical systems which do not yield to simplistic treatments if one seeks them to be relevant. Amongst such topics, *sustainability* is of particular importance for the future of society and organized humanity. Molina-Perez et al. bring together an arsenal of methods to address the challenges of such models. An earlier model for the interplay of economical and ecological parameters, solved by constrained optimization, is considered under different policy regimes, uncertain stressors, and multiple experimentation with different elasticity parameters; it is subjected to machine-learning based clustering analysis for the parameters that produce stable vs. unstable regimes. All components, complex modeling, optimization, machine learning, and data mining work together to obtain a picture not only of how the system behaves on the whole, but also what policies should be enacted to increase the chance of obtaining desirable results.

Grisogono discusses how artificial intelligence (AI) could help tackle global complex problems, such as climate change, collapsing ecosystems, international conflicts, extremism, public policy, economics, and governance. These problems require decision-making to attempt solutions or improvements. AI has the potential of mitigating failures and limitations in human decision-making, leading to a balance between robustness and adaptability. Nevertheless, there are also risks and drawbacks in AI, so its proper use and development should be discussed in detail.

Our research topic also shows how the concepts of complexity and self-organization are helpful in studying biological systems. Two above-mentioned papers Morales and Froese and Ramos-Fernandez et al. have contributions to both cognitive science and biology.

Our understanding of morphogenesis, as an important process of natural development, has advanced recently in part because of computing power, data availability, and algorithms. Thus, Pastor-Escuredo and del Álamo explore how computation is contributing to developmental biology. Computational models and simulations are proving useful to unravel the dynamic and multi-scale nature of morphogenesis. Machine learning and in particular deep learning architectures are promising in this respect. There are also potential applications for tissue engineering, identification of therapeutic targets, and synthetic life.

Finally, Casiraghi and Schweitzer address questions related to **computational social science**. In particular, they propose a method for improving the robustness of online social networks. Their aim is to prevent drop-out cascades of users. This is done using strategies to influence particular agents, reducing their probability of leaving the network, and thus considerably reducing drop-out cascades and increasing robustness.

As topics, complexity and self-organization have worked their way out of an exotic niche into the center of human activities. In contrast to the traditional reductionist treatment of scientific investigations, in today's crosstalk of disciplines, there is no field of human endeavor or study that can be considered in isolation. Understanding complexity has become a crucial skill in studying how the interacting levels of organismic function, society, ecological, and economical webs lead to a functioning whole—or to its disintegration. The richness of the contributions to this research topic serves as a showcase for the width and variety of tools and viewpoints that are being marshaled to this purpose.

## Author Contributions

All authors listed have made a substantial, direct and intellectual contribution to the work, and approved it for publication.

## Conflict of Interest

The authors declare that the research was conducted in the absence of any commercial or financial relationships that could be construed as a potential conflict of interest.

